# HnRNPK/miR-223/FBXW7 feedback cascade promotes pancreatic cancer cell growth and invasion

**DOI:** 10.18632/oncotarget.15529

**Published:** 2017-02-20

**Authors:** De He, Cheng Huang, Qingxin Zhou, Dawei Liu, Longhui Xiong, Hongxia Xiang, Guangnian Ma, Zhiyong Zhang

**Affiliations:** ^1^ Department of General Surgery, The Affiliated Baoan Hospital of Southern Medical University, Shenzhen, Guangdong, 518101, China; ^2^ Guangdong Medical University Graduate School, Zhanjiang, Guangdong, 524001, China; ^3^ Department of Gastrointestinal Oncology, Cancer Hospital of Harbin Medical University, Harbin, Heilongjiang, 150086, China; ^4^ Department of Surgery, Robert-Wood-Johnson Medical School University Hospital, Rutgers University, New Brunswick, NJ 08901, USA

**Keywords:** HnRNPK, miR-223, FBXW7, pancreatic ductal adenocarcinoma, GSK3

## Abstract

Several studies have identified miR-223 critically involved in various types of cancer, including pancreatic ductal adenocarcinoma (*PDAC*). However, its action and regulatory mechanisms in PDAC remains largely unclear. In this study, we found that the expression levels of miR-223 were increased in clinical samples with PDAC (81.6%). The upregulation of miR-223 increases the proliferation, migration, and invasive abilities of PDAC cells *in vitro* and *in vivo*. Mechanistically, miR-223 directly targeted FBXW7 and overexpression of FBXW7 reverted miR-223- induced drastic proliferation in PDAC cells. Interestingly, miR-223 promoter was found to form a coprecipitable complex with hnRNPK, and siRNA knockdown of hnRNPK in PDAC cells reduced the levels of miR-223. These results show that hnRNPK is a cellular protein that binds and affects the accumulation of miR-223 in PDAC. Furthermore, FBXW7 interacts with hnRNPK and promotes its degradation, which requires phosphorylation of hnRNPK at threonine 1695 by GSK3. Consistently, we observed an inverse expression pattern between FBXW7 and miR-223, whereas a positive expression pattern between miR-223 and hnRNPK was found in human PDAC tissues. These data unveiled an important new miR-223/FBXW7/HnRNPK feedback cascade in human PDAC.

## INTRODUCTION

Pancreatic ductal adenocarcinoma (PDAC) is the most common type of pancreatic malignancy and has with a 5-year survival of less than 5% [1]. This poor survival rate has not improved in the last decades. One of the reasons of this dismal prognosis is individual cancer cells detaching from the primary tumor, migrating to the blood/lymph, and colonizing distant organs or tissues [2]. Dysregulation of signaling pathways, and abnormal expression and function of some molecules are often related to cancer metastasis [3]. Therefore, deciphering the potential mechanisms underlying PDAC invasion and metastasis is of paramount importance and likely to contribute to the development of effective therapeutics for treating PDAC patients. Another reason of this dismal prognosis is the lack of sensitive and specific biomarkers for PDAC, which adds to the existing problems of increasing incidence and poor prognosis of this lethal disease, because early detection of pancreatic intraepithelial neoplasia or mucinous neoplasms would be the best option to improve patient survival. Thus, there is an immediate need to identify new targets for the treatment of PDAC in general and this aggressive subtype in particular.

MicroRNAs (miR) are post-transcriptional regulators with tumor-suppressive or oncogenic roles in various carcinomas [4]. Recent studies have demonstrated that miRNAs function as potential biomarkers for early detection of various cancers, including PDAC [5]. MiRNAs are small (19–24 nucleotides) non-coding RNAs playing an important gene-regulatory role in the post-transcriptional phase [5]. Given that miRNAs are stable in body fluids, either in secreted microvesicles or bound to carrier proteins, this offers a unique opportunity to exploit miRNAs as potential biomarkers for early detection of various cancers, including PDAC [6]. Although large numbers of miRNAs such as miR-223 have been found to be associated with human PDAC [7], less is known about their regulatory mechanisms.

The heterogeneous nuclear ribonucleoprotein (hnRNP)K is an essential RNA and DNA binding protein involved in gene expression and signal transduction including DNA transcription, RNA splicing, RNA stability and translation [8]. The role of hnRNPK in PDAC is relatively understudied. However, several cellular functions strongly indicate that hnRNPK is involved in tumorigenesis. The altered protein expression and the subcellular distribution of the hnRNPK protein in PDAC lead to silenced mRNA translation of tumor suppressor genes and thus contribute to PDAC development [9]. These reports indicate that hnRNPK is an attractive therapeutic target of PDAC. It is well-known that hnRNP K associates with different kinds of proteins to execute their functional roles [10]. This raises the question of whether hnRNPK could also interact with miR-223 in a manner similar to that of protein-coding genes. In addition, very little was known about how the expression of hnRNPK was regulated in PDAC?

In the present study, we have for the first time investigated the crosstalk between hnRNPK protein and miR-223/FBXW7 axis in PDAC, which contributes to PDAC pathogenesis. We identified FBXW7 not only as a major downstream target of hnRNPK-miR-223 complex driving the tumorigenicity of poorly differentiated PDAC, but also is a E3 ligase of hnRNPK. Thus, our results provide novel insights into the epigenetic machinery in PDAC and suggest therapeutic strategies for this malignancy.

## RESULTS

### MiR-223 expression is up-regulated in PDAC tissues and cell lines, whichcorrelates with the poor prognosis of PDAC

**Figure 1:**
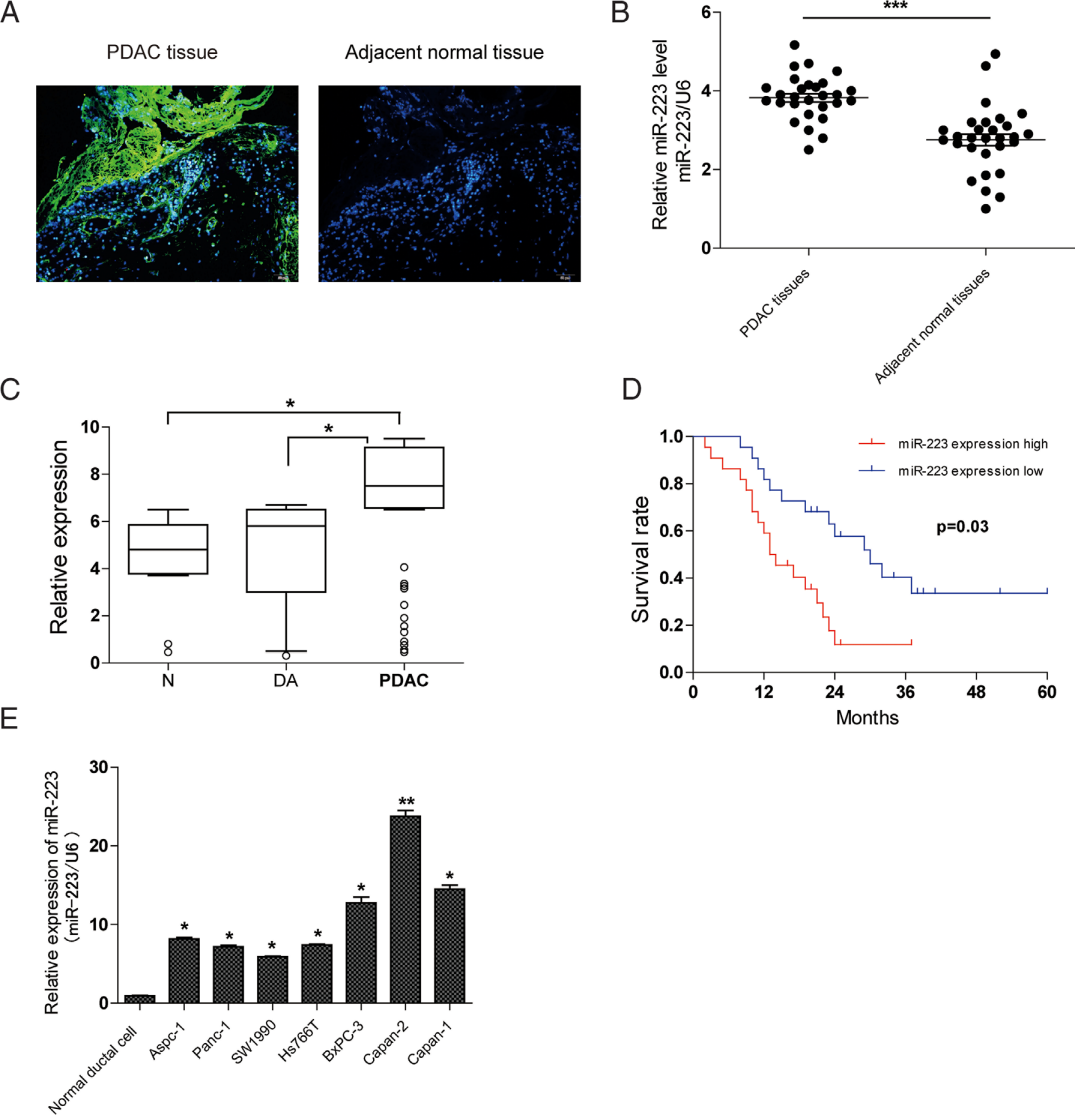
Expression and diagnostic significance of miR-223 in PDAC **A**. Positive staining for miR-223 was found in human PDAC tissues but not in adjacent normal pancreatic tissues by ISH. **B**. qRT-PCR analysis of miR-223 levels in 31 pairs of PDAC tissues and adjacent non-cancer tissues. **C**. qRT-PCR analysis of serum miR-223 levels in PDAC patients (n=60), 40 age-matched normal (N) controls, and 17 duodenal adenocarcinoma (DA). The relative miRNA expression level was determined by normalizing against U6 snRNA. **D**. Kaplan–Meier survival analysis of PDAC patients grouped by the expression levels of miR-223. **E**. MiR-223 expression in seven PDAC cell lines versus normal ductal cell.

To decide the functional role of miR-223 in PDAC, we utilized ISH to study the expression pattern of miR-223 in cancer and corresponding non-cancer tissues. Positive cytoplasmic staining for miR-223 was evident in cancer cells, whereas no miR-223 positivity was found in the adjacent normal pancreatic tissues (Figure 1A). To confirm the ISH results, miR-223 expression in 31 pairs of PDAC tissues and adjacent normal tissues was examined by qRT-PCR analysis. As shown in Figure 1B, compared to adjacent normal tissues, miR-223 expression was significantly enhanced in PDAC tissues. In addition, qRT-PCR analysis revealed that PDAC patients had > 1.5-fold greater concentrations of serum miR-223 than duodenal adenocarcinoma patients or healthy controls (*P* <0.05 for each comparison), whereas no significant difference in the serum miR-223 level was observed between duodenal adenocarcinoma patients and healthy controls (Figure 1C). We next investigated whether miR-223 expression correlated with the prognosis of PDAC. Kaplan–Meier analysis demonstrated that the median survival time of PDAC patients with low and high expression of miR-223 was 31.4 and 14.6 months, respectively (Figure 1D), which is consistent with analysis about the relationship between miR-223 expression and clinicopathologic parameters in 60 PDAC patients (Table 1). We also found that miR-223 was relatively higher in a series of tested human PDAC cell lines as compared with that found in normal human pancreatic duct epithelial cells (Figure 1E). Together, these data suggest that miR-223 might be an oncogenic miRNA in PDAC.

**Table 1:** The relationship between miR-223 expression and clinicopathologic parameters in 60 PDACs

Factor	Characteristic	miRNA-223 expression(case)		P-values
		Higher	Lower	
**Gender**	Men	23	15	*p*=0.651
	Women	12	10	
**Age(years)**	<=59	20	14	*p*=0.407
	>59	18	8	
**Tumor size**	<=4	28	13	*p*=0.695
	>4	12	7	
**Tumor site**	Head	20	12	*p*=0.988
	Head process	11	7	
	Body and tail	6	4	
**Histologic grade**	G1, G2	5	40	*p*=0.012*
	G3	9	6	
**pT category**	T1, T2	9	12	*p*=0.004**
	T3, T4	31	8	
**Lymph node metastasis**	Yes	14	9	*p*=0.009**
	No	33	4	

^*^p<0.05, **p<0.01, Chi-square test.

### HnRNPK associates with miR-223 promoter and transcriptionally upregulates its expression

**Figure 2:**
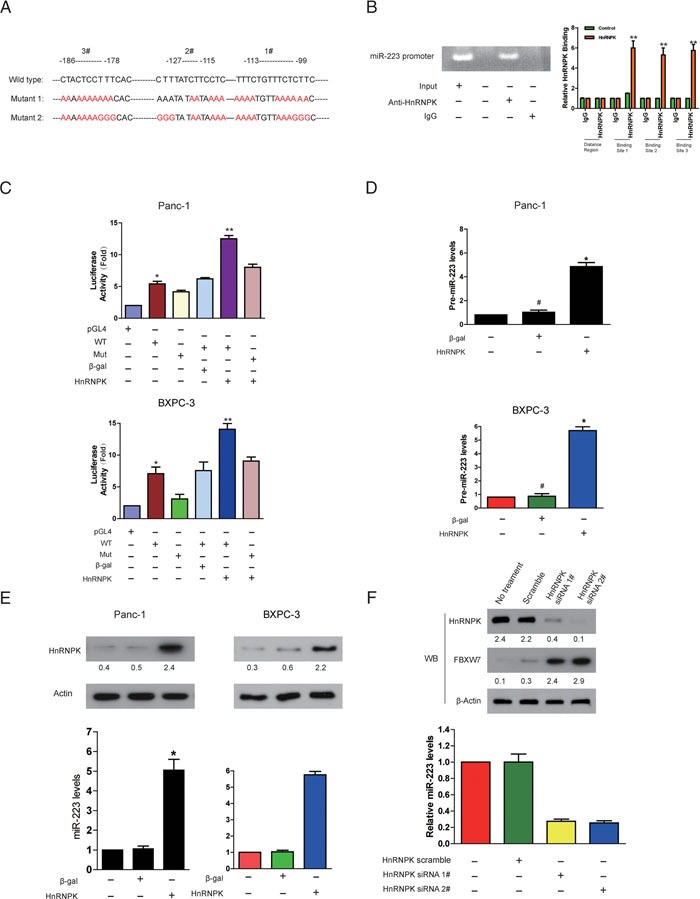
HnRNPK directly binds to miR-223 promoter and transcriptionally stimulates its expression **A**. MiR-223 promoter region contains three potentially conserved hnRNPK binding sites. The sequences were then mutated to disrupt hnRNPK binding to the miR-223 promoter sequences as indicated. **B**. ChIP analysis of hnRNPK binding to the promoter of miR-223 in PDAC cells. Panc-1 and BxPC-3 cells were collected for ChIP assay. IgG was served as negative control. **C**. Firefly reporter activity measured from Panc-1 cells infected with adenoviruses expressing hnRNPK, or β-galactosidase (β-gal), or empty vector (pGL4), or with constructs containing the wild-type (WT) miR-223 promoter, or the miR-223 promoter mutated at putative hnRNPK binding sites (Mut). The renilla was used as internal control. **D** and **E**. Enforced expression of hnRNPK induces an elevation of pre-miR-223 and miR-223 levels. Panc-1 and BxPC-3 cells were infected with adenoviral hnRNPK or β-gal. pre-miR-223 (D) and miR-223 (E), were detected by qRT-PCR. *p <0.05*, N=6. The results were normalized to that of U6. **F**. Knockdown of hnRNPK reduces miR-223 expression while increases the level of FBXW7. BxPC-3 cells were infected with adenoviral hnRNPK siRNA or its scramble. 24 hours after infection, miR-223 was detected by qRT-PCR. HnRNPK and FBXW7 were analyzed by immunoblot. The indicated proteins in (D-F) were detected with WB and quantified with NIH ImageJ software (Bethesda, MA).

Although we and other groups found that miR-223 expression was significantly enhanced in different types of tumor tissues including PDAC [7, 13], very little is known about the mechanisms underlying the transcriptional upregulation of miR-223 in PDAC. To search for novel transcription factor of miR-223, we analyzed the promoter region of miR-223, and observed that miR-223 promoter region contains three CT-rich DNA sequences, which is termed the CT element [14] (Figure 2A). Given that hnRNPK has been shown to transactivate the CT element *in vivo* and mediate the activity of the CT element *in vitro* [15], we hypothesized that hnRNPK transcriptionally upregulates the expression of miR-223 in PDAC. To verify this idea, experiments were performed to define the CT element binding properties of hnRNPK. Figure 2B demonstrated an association of hnRNPK with miR-223 promoter using ChIP analysis. Luciferase assay demonstrated that hnRNPK stimulates the promoter activity of the wild type miR-223 but not that of the mutated form (Figure 2C). We further tested whether hnRNPK can regulate the level of miR-223 expression. Enforced expression of hnRNPK was found to elevate both pre-miR-223 (Figure 2D) and mature miR-223 levels (Figure 2E). Conversely, knockdown of hnRNPK reduced miR-223 levels in PDAC cells (Figure 2F). Together, these results suggest that hnRNPK transcriptionally promotes miR-223 expression.

### MiR-223 promotes cancer cell proliferation and migration in PDAC

**Figure 3:**
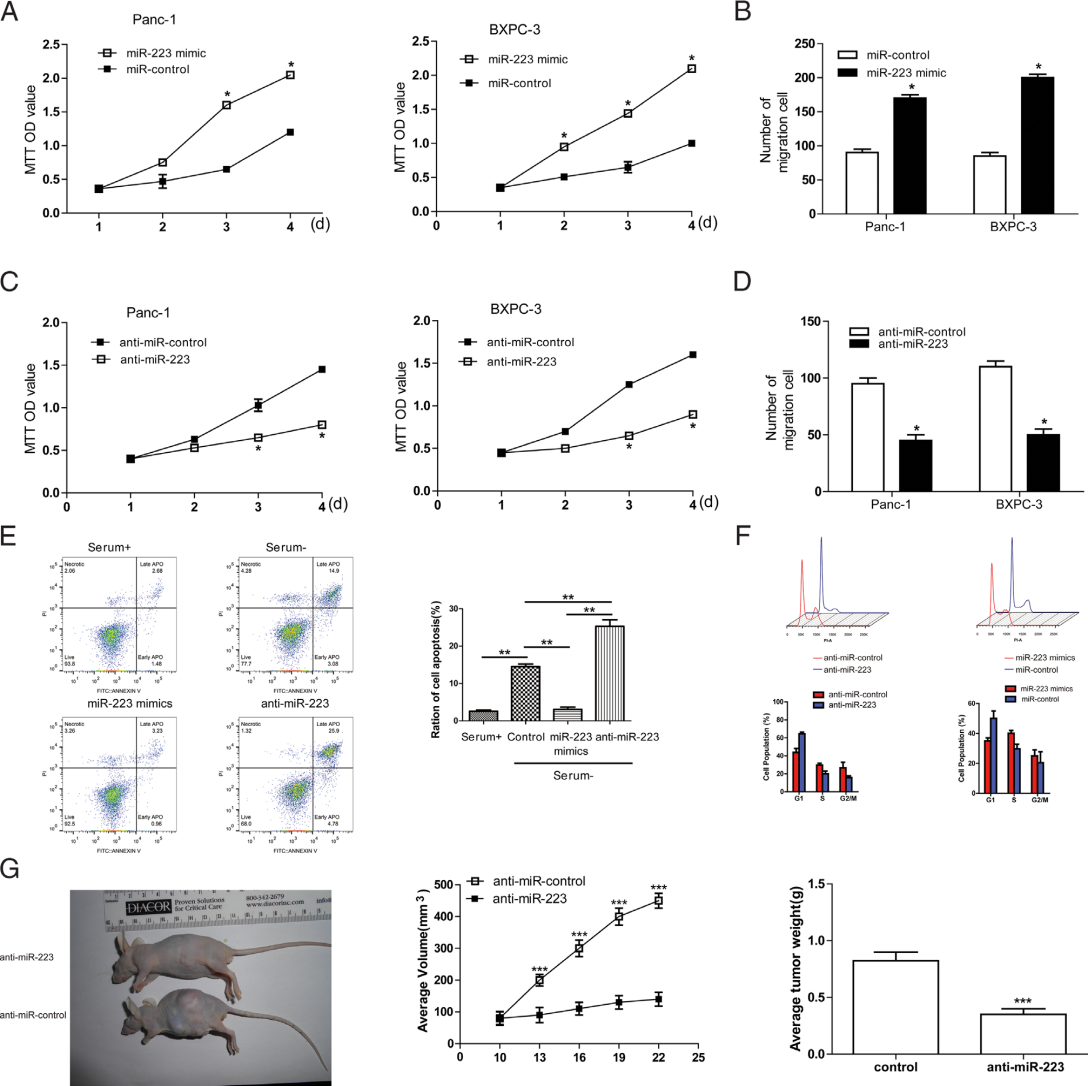
miR-223 promotes PDAC cell growth and migration, while inhibits apoptosis and enhances S phase of PDAC cells **A**. Cell viability was measured using the MTT assay in Panc-1 and BXPC-3 cells transfected with miR-223 mimics or miR-control (A) and cell migration was assessed using the Transwell assay **B., C** and **D**. Panc-1 and BXPC-3 cells were transfected with antisense oligonucleotides against miR-223 (miR-223 inhibitor), or anti miR-control, and cell viability was assessed using the MTT assay (C), and cell migration was assessed using the Transwell assay (D). **E**. miR-223 suppresses cell apoptosis (n = 5). **F**. The cell cycle profiles of indicated cells determined by flow cytometry assays. Percentages of different cell cycle phases were presented. **G**. Knockdown of miR-223 inhibited the growth of PDAC cells-engrafted tumors. Representative tumors were photographed at 15 days after the first treatment with miR-223 inhibitor or control (left panel). Graph representing tumor volumes (middle panel) and tumor weight averages (right panel) between control and miR-223 inhibitor-treated mice groups (n=8 per group) at the end of the experiment were presented.

To examine the effect of miR-223 expression on cell viability, PDAC cells were transfected with miR-control, miR-223 mimics or miR-223 inhibitor, and analyzed using the MTT and Transwell migration assays. Relative levles of miR-223 were confirmed by qRT-PCR (Supplementary Figure 1). Ectopic expression of miR-223 in the Panc-1 and BxPC-3 cell lines significantly increased cell viability in a time-dependent manner (Figure 3A) and increased migratory ability (Figure 3B) compared to miR-control transfected cells. Conversely, inhibition of miR-223 significantly caused the opposite phenotypes (Figure 3C and 3D). We also determined the apoptosis levels of Panc-1 cells. As expected, ratio of cell apoptosis significantly increased in FBS-free medium, whereas it was inhibited with the transfection of miR-223 mimics (Figure 3E). In addition, we examined the effect of miR-223 on cell cycle progression. Compared with miR-control, miR-223 mimics resulted in an enhanced percentage of S phase in PDAC cells, whereas miR-223 inhibitors caused a G1 cell-cycle arrest, which was evidenced by the reduced percentage of S and the increased percentage of G1 (Figure 3F). Importantly, an *in vivo* tumor formation assay demonstrated that miR-223 inhibitor inhibited the growth of PDAC cells-engrafted tumors compared to control oligonucleotides-treated tumors (Figure 3G). Collectively, these data clearly demonstrate that miR-223 has a growth-promotive function in PDAC.

### The effect of miR-223 on pancreatic cancer cell viability and invasion is mediated by the down regulation of FBXW7

**Figure 4:**
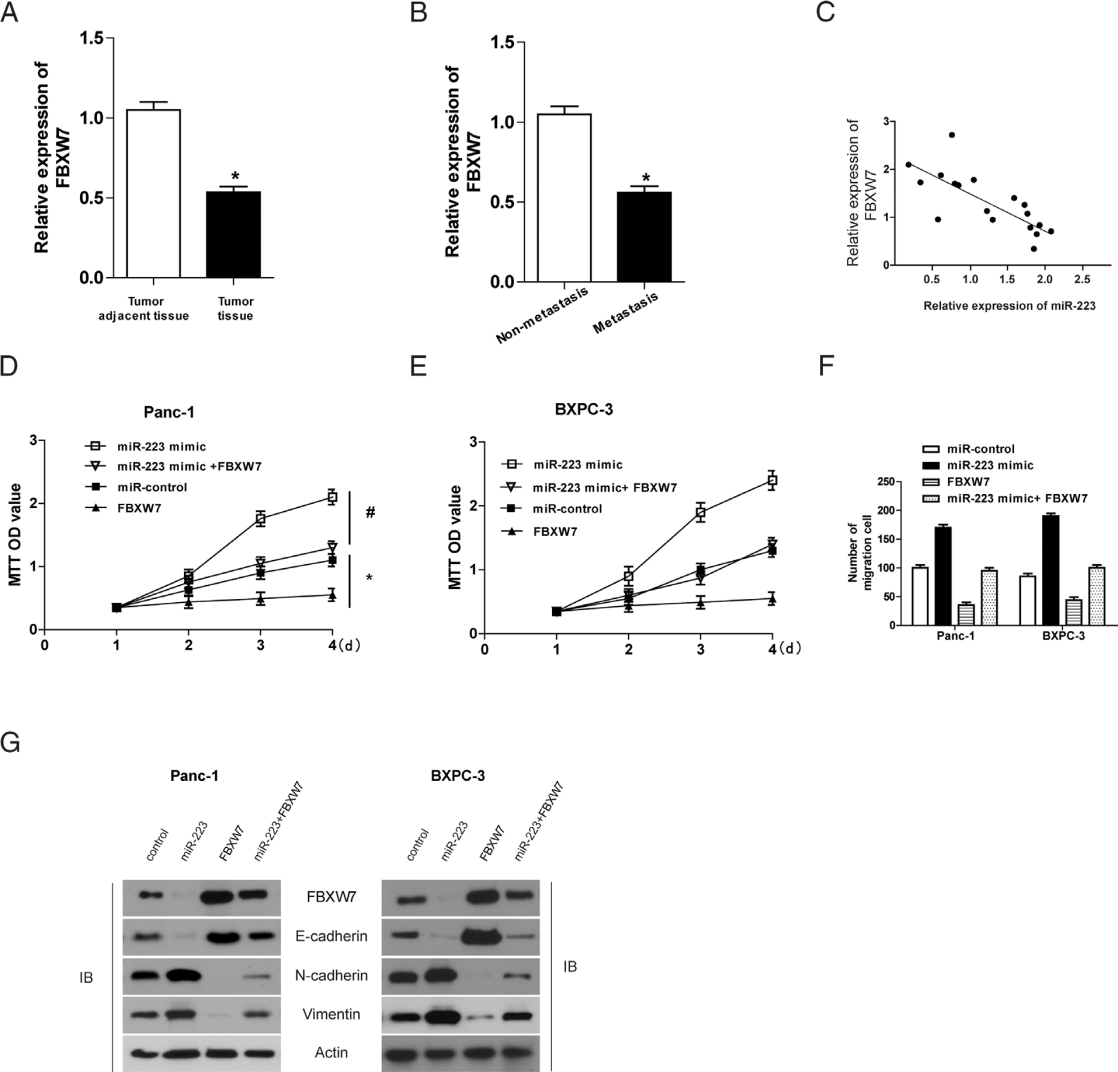
FBXW7 overexpression rescues the effect of miR-223 on PDAC cell proliferation and migration **A** and **B**. The level of FBXW7 was analyzed in human pancreatic cancer and adjacent non-tumor tissues (A) and in metastasis and non-metastasis associated tissue samples (B) by qRT-PCR. **C**. Correlation between miR-223 and FBXW7 protein expression in tumor samples. Panc-1 and BXPC-3 cells overexpressing miR-223, FBXW7, or both was determined using the MTT assay **D** and **E**. and migration was assessed using the Transwell assay (*P* <0.05, N=5) **F., G**. The expression of EMT markers was assessed by western blotting in miR-223 or FBXW7 expressing cells.

Given that miR-223 functions as an oncogene in human gastric cancer by targeting FBXW7 [16], and that genistein treatment significantly inhibited miR-223 expression but up-regulated FBXW7 in PDAC cells [7], we further examine the involvement of FBXW7 in the effect of miR-223 in human patients with pancreatic cancer. qRT-PCR showed that compared to adjacent non-tumor tissues, FBXW7 was significantly down regulated in tumor tissues (Figure 4A), and also significantly decreased in metastatic compared to non-metastatic tissues (Figure 4B). Figure 4C shows an inverse correlation between FBXW7 and miR-223 expression in pancreatic tumors. Importantly, over-expression of FBXW7 decreased the viability and migratory ability of Panc-1 and BxPC-3 cells in a time dependent manner, and reversed the miR-223-induced increase of cell viability and migration (Figure 4D, 4E, and 4F). Overexpression of FBXW7 also up-regulated the epithelial marker E-cadherin and down-regulated the mesenchymal markers N-cadherin and vimentin, and reversed the miR-223-mediated induction of EMT in Panc-1 and BxPC-3 cells (Figure 4G). Taken together, our data suggest that the impact of miR-223 on cell proliferation, migration and EMT is mediated by the down -regulation of its target FBXW7 in PDAC cells.

### HnRNPK is a substrate of the FBXW7 E3 ligase and interacts with FBXW7 through a conserved degron sequence phosphorylated by GSK3β and Erk1

**Figure 5:**
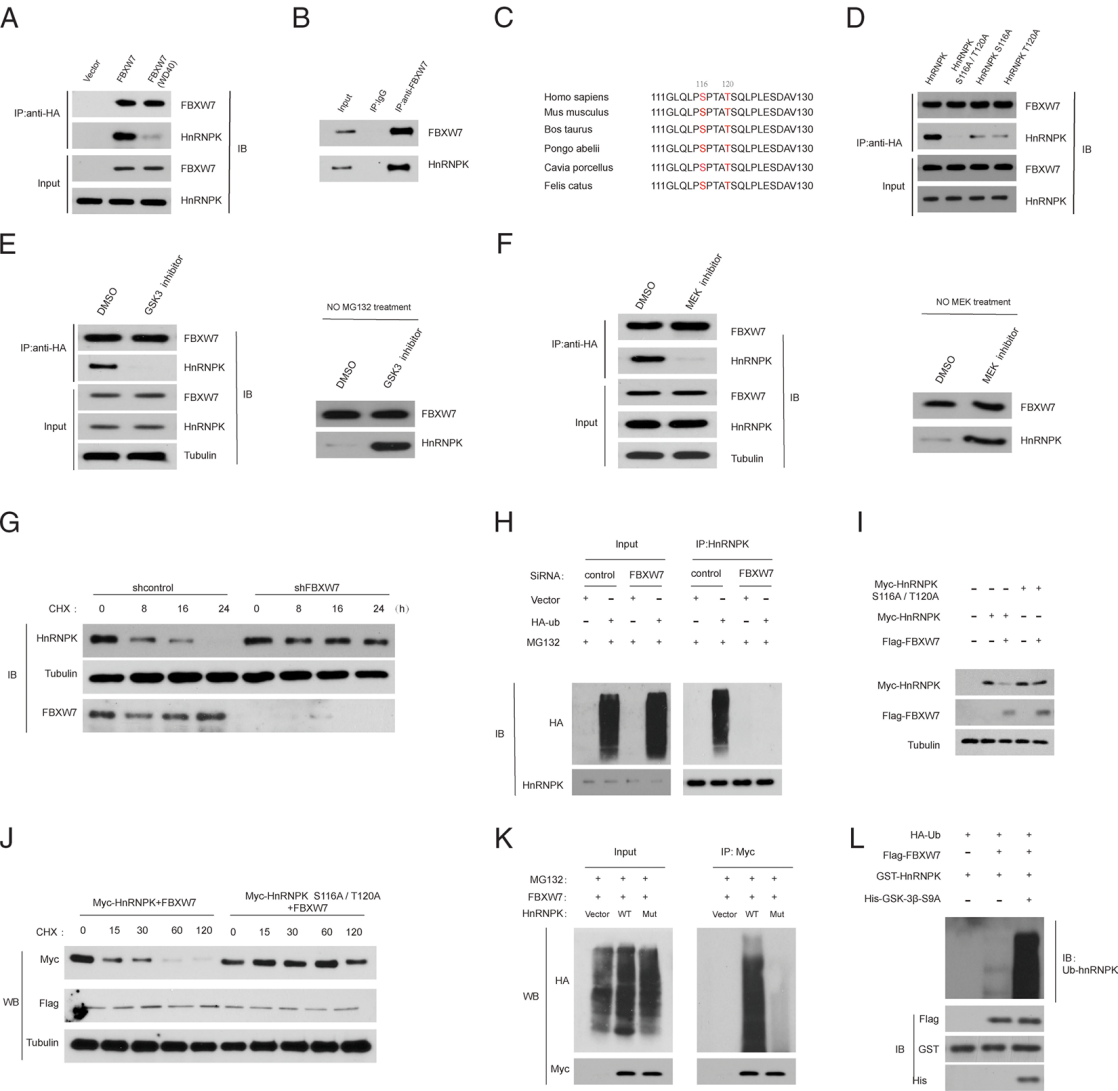
HnRNPK is a substrate of the FBXW7 ubiquitin ligase **A**. FBXW7 binds to hnRNPK through specific residues in the WD40 domain. HEK293T cells were transfected with constructs encoding FLAG-tagged-hnRNPK, and FLAG–HA-tagged empty vector, or FLAG–HA-tagged-FBXW7 or FLAG–HA-tagged-FBXW7 (WD40), a substrate-binding mutant, in which three residues within one of the seven WD40 repeats of FBXW7 have been mutated. HA-tagged-FBXW7 was immunoprecipitated (IP) from cell extracts with anti-HA resin, followed by immunoblotting as indicated. **B**. Native FBXW7 was immunoprecipitated (IP) from SW1990 cell extracts with anti-FBXW7 antibody, followed by immunoblotting as indicated. Rabbit IgG was used as control. **C**. Alignment of the human hnRNPK protein region containing the putative degron with hnRNPK from various organisms. Conserved phosphoamino acids (amino acids 116 and 120 in the human sequence) are highlighted. **D**. Both Ser-116 and Thr-120 in hnRNPK are required for the interaction with FBXW7. HEK293T cells were transfected with FLAG–HA-tagged FBXW7 and constructs encoding FLAG-tagged hnRNPK or hnRNPK (S116/T120A) or hnRNPK (S116A) or hnRNPK(T120A). HA-tagged FBXW7 was immunoprecipitated (IP) from cell extracts with anti-HA resin, followed by immunoblotting as indicated. **E**. Interaction between hnRNPK and FBXW7 and the degr- adation depend on GSK3β activity. HEK293T cells were transfected with constructs encoding FLAG-tagged hnRNPK and FLAG–HA-tagged FBXW7. Cells were treated with GSK3i IX (10 μM for 10 h) or dimethylsulphoxide (DMSO). HA-tagged FBXW7 was immunoprecipitated (IP) from cell extracts with anti-HA resin, followed by immunoblotting as indicated. **F**. Interaction between hnRNPK and FBXW7 and the degradation of hnRNPK depend on Erk1 activity. HEK293T cells were transfected as described in (E). Cells were treated with MEK1 inhibitor U0126 (10 μM for 2 h) or DMSO. **G**. The indicated cells line were exposed to CHX (10 μg/ml) and then harvested at the different time points as indicated for western blot. The indicated proteins were detected with WB and quantified with NIH ImageJ software (Bethesda, MA). HnRNPK levels were normalized to tubulin and plotted as the relative hnRNPK levels compared with those at time 0 of CHX treatment. **H**. 48 h after transfection with control or FBXW7 siRNA, SW1990 cells were further transfected with HA-ubiquitin or empty vector for an additional 48 h, and then treated with 10 μm MG132 for 2 h. The lysates were immunoprecipitated with an anti-hnRNPK antibody, and ubiquitinated hnRNPK (Ub-hnRNPK) was determined using an anti-HA antibody. **I**. 293T cells were co-transfected with FLAG-FBXW7 and myc-hnRNPK or myc- hnRNPK (S116A/T120A) mutant for 48 h, and then harvested for subsequent co-IP. **J**. 293T cells were co-transfected as in (I), and 48 h later treated with 10μg/ml CHX. At the indicated time points, the cells were harvested for preparation of western blot. **K**. 293T cells were co-transfected as in (I), and after 48 h, cells were transfected with an empty vector or HA-ubiquitin (HA-Ub). After another 48 h, cells were treated with 10 μm MG132 for 2 h and then subjected to co-IP to detect ubiquitinated hnRNPK. **L**. *In vitro* ubiquitination assay. Recombinant HnRNPK was incubated with recombinant FBXW7. HA-ubiquitin and active GSK3-S9A were added to the indicated reaction.

Given that FBXW7 is an ubiquitin ligase [17], we wondered whether hnRNPK is its substrate. Interestingly, hnRNPK was detected in FBXW7 immunoprecipitates (Figure 5A). However, a WD40 domain mutant FBXW7 which lacks the ability to bind protein substrates [18] significantly reduced its interaction with hnRNPK (Figure 5A). Similarly, we observed that endogenous FBXW7 bind to hnRNPK (Figure 5B). Alignment of the hnRNPK protein sequence indicated the presence of one evolutionarily conserved amino-acid sequences resembling the canonical FBXW7 degradation motif (degron) S/TPXXS/T [17] (Figure 5C). Interestingly, an hnRNPK mutant containing alanine substitutions at both Ser-116 and Thr-120 [hnRNPK (S116A/T120A)] failed to bind FBXW7 (Figure 5D), suggesting both Ser-116 and Thr-120 as necessary residues contributing to its interaction with FBXW7.

The most common mechanism that controls destruction of FBXW7 substrates is phosphorylation of a specific phosphorylated sequence motif [18]. We found that treatment of cells with a GSK3 inhibitor or a MEK inhibitor (U0126) markedly decreased the affinity of FBXW7 for hnRNPK and inhibited the degradation of hnRNPK (Figure 5E and 5F). Moreover, depletion of FBXW7 increased the half-life of hnRNPK protein (Figure 5G). And hnRNPK polyubiquitylation was markedly increased with FBXW7 overexpression, whereas induced hnRNPK polyubiquitylation was significantly reduced on FBXW7 silencing (Figure 5H), suggesting that FBXW7 binds and directly controls hnRNPK ubiquitylation.

Finally, we wanted to determine whether phosphorylation of hnRNPK on Ser-116 and Thr-120 affects its ubiquitylation. When co-expressed with FBXW7, the levels of WT-hnRNPK were clearly reduced, while the hnRNPK (S116A/T120A) mutant (Mut) levels were not decreased at all (Figure 5I). We also found that hnRNPK (S116A/T120A) was degraded much more slowly than WT hnRNPK (Figure 5J), indicating that hnRNPK (S116A/T120A) mutant is much more stable than WT hnRNPK. These findings indicate that hnRNPK (S116A/T120A) mutant is resistant to FBXW7-mediated degradation. Moreover, the ubiquitination of hnRNPK (S116A/T120A) mutant was also substantially reduced in comparison with that of WT hnRNPK (Figure 5K). *In vitro* ubiquitination assay further confirmed the above findings (Figure 5L). Collectively, FBXW7 is a novel unrecognized E3 ligase of hnRNPK in PDAC cells.

### Increased miR-223 expression is correlated with decreased FBXW7 and enhanced hnRNPK expression in human PDAC specimens

**Figure 6:**
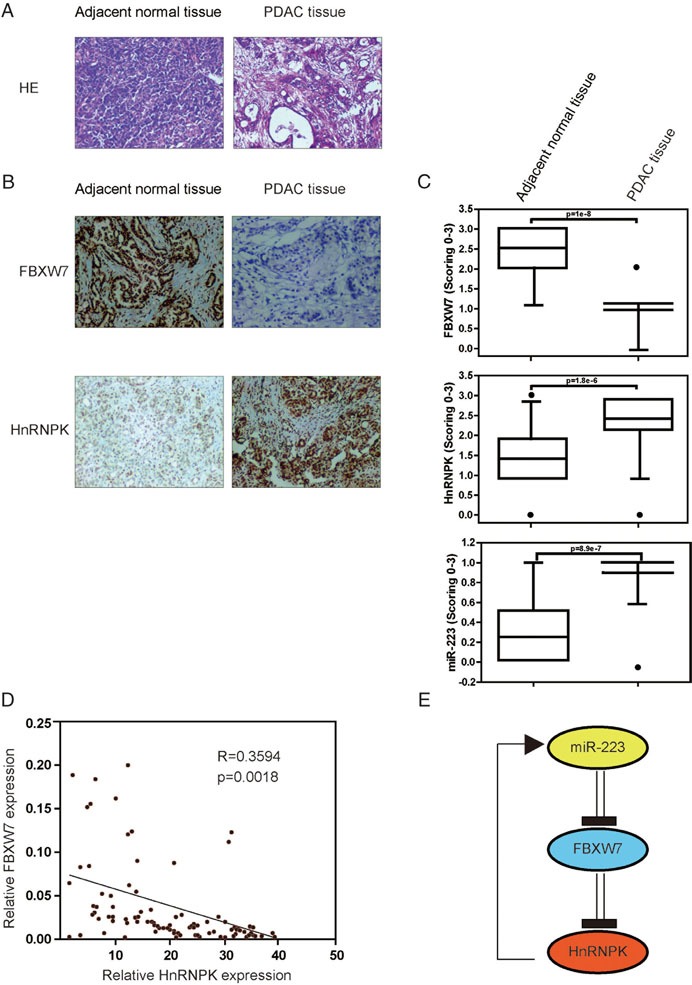
Low FBXW7 is tightly correlated with higher miR-223 and hnRNPK levels in human PDAC tissues **A**. Hematoxylin and eosin (HE) staining representative histology of PDAC and non-PDAC specimens. **B**. Representative slides of IHC staining of FBXW7 and hnRNPK, and *in situ* hybridization (ISH) probing of miR-223 in PDAC and normal pancreatic tissue tissues (left panel). A scrambled probe for miR-223 was used as a negative control that did not stain any tissue sections. **C**. Expression levels of FBXW7 and hnRNPK, and miR-223 were scored in PDAC (*n*=60) and normal pancreatic tissue tissues (*n*=20). The gradation was scored from 0 to 3 according to the intensity of staining (0, negative; 1, weak; 2, moderate; 3, strong). Whisker box plot with boxes indicate 25th and 75th percentile; thick lines, the mean values; whisker caps, 10th and 90th percentile; filled circle, outliers. *P*-values of two-tailed Student's *t*-test are provided. We found miR-223 expression was significantly increased in PDAC specimens (0.89±0.28) compared with non-PDAC control specimens (0.25±0.41; *P*=8.9e−7), whereas FBXW7 was found to be decreased, and hnRNPK expression was subsequently increased compared with non-PDAC specimens. **D**. Correlation between FBXW7 and hnRNPK expression in the clinical samples. FBXW7 is inversely correlated with hnRNPK in PDAC. **E**. Schematic diagram of the hnRNPK/miR-223/FBXW7 feedback loop.

Confident with the *in vitro* data, we further studied the correlation of miR-223, FBXW7 and hnRNPK in human PDAC samples. A total of 60 PDAC specimens along with 20 controls (Figure 6A) were analyzed for FBXW7 and hnRNPK levels by IHC and miR-223 levels by ISH (Figure 6B). We found a significant decrease of FBXW7 expression in PDAC specimens compared with controls. As expected, the expression levels of miR-223 and hnRNPK were significantly increased in PDAC specimens compared with those in controls, which were consistent with the data from the xenograft mouse model. Moreover, there was an inverse relationship between FBXW7 and hnRNPK expression in PDAC specimens and controls respectively (Figure 6C). These clinical data further support mechanism postulating that miR-223/FBXW7/hnRNPK feedback loop promotes PDAC growth and metastasis (Figure 6D).

## DISCUSSION

In the current study, we demonstrate that up-regulation of miR-223 is potentially responsible for aberrant expression of hnRNPK in PDAC tumor cells. HnRNPK in turn promotes PDAC tumorigenesis by regulating miR-223 transcription and decreasing FBXW7 expression, which requires phosphorylation of hnRNPK at threonine 1695 by GSK3. Our findings collectively show an important new miR-223/FBXW7/HnRNPK feedback loop. For the first time, this provides a mechanistic explanation for the correlation between miR-223/FBXW7 and hnRNPK in pancreatic cancer. Importantly, high-level hnRNPK expression was found to be correlated with poor prognosis and increased expression of miR-223 in PDAC patients. Therefore, our study paves the way for further understanding of the complex biology involved in PDAC cancer progression.

Overexpression of hnRNPK is found in various cancers and has been correlated with poor prognosis, however, its action mechanism in tumor development and progression are largely under-explored [9]. Recent work has shown that hnRNPK regulates antiapoptosis and cell migration [19]. And FLIP, FGF2, SULF2 and MMP13 are known to be overexpressed in various cancers and downstream of hnRNPK [20]. Consistent with these findings, in the current study, we show a new function for hnRNPK in miR-223 transcriptional activation, although miR-223 had not been previously reported as hnRNPK targets. Moreover, induction of miR-223 by hnRNPK decreases the level of FBXW7 in PDAC cells. Although hnRNPK was shown to increase the stability of mature miR-122 possibly by protecting a nonbase-paired portion of miRNA [21], our results underscore a previously uncharacterized role for general RNA-binding protein as a transcription factor that facilitates the expression of specific miRNA. Therefore, hnRNPK may be highlighted as a novel agent for the treatment of patients with PDAC.

Notably, hnRNPK has been known to be a substrate of the ubiquitin E3 ligase MDM2, and upon DNA damage, is de-ubiquitylated [22], however, the ways in which hnRNP K is itself regulated remain largely obscure in other conditions. Here, our findings establish hnRNPK as a new FBXW7 target. It has been suggested that the interaction between FBXW7 and substrate requires prior phosphorylation of substrates within their CPDs, and that this phosphorylation is often mediated by GSK3 [23]. It has also been suggested that hnRNPK can be phosphorylated at multiple sites, and phosphorylation at a given site of hnRNPK may affect its biological functions [24]. For example, Erk-mediated phosphorylation of hnRNPK increases stability of hnRNPK protein-bound transcripts and also drives hnRNPK protein export from the nucleus [25]. Indeed, the present study provides robust evidence indicating that hnRNPK is degraded through a phosphorylation-dependent and FBXW7-mediated ubiquitination/proteasome pathway based on the following findings; 1) hnRNPK is regulated by ubiquitination/proteasome-mediated degradation; 2) hnRNPK degradation is mediated by FBXW7; 3) a functional CPD motif is present in hnRNPK; 4) hnRNPK degradation is GSK3-dependent; 5) GSK3 directly phosphorylates the hnRNPK CPD motif at Thr-1695. Thus, the current findings provide another piece of evidence supporting the involvement of protein degradation in the regulation of hnRNPK signaling. Whether hnRNPK is regulated through a similar mechanism in other cancers needs further evaluation. In addition, FBXW7 is a tumor suppresser that is frequently mutated in certain types of human cancer, but the exact mechanisms underlying its tumor suppressive function remain unclear [26]. Although FBXW7 appears to be associated with regulation of the degradation of multiple oncogenic proteins such as c-Myc, cyclin E, and c-Jun [26], our findings presented in this study may suggest a putative tumor-suppressive mechanism of FBXW7 involving negative regulation of hnRNPK stability. Hence further study in this direction is warranted.

As indicated in many papers, the same miR might play a different role in various cancers (read the following references), such as miR-146a acts as oncomiR or tumor suppressor in different tumors [27, 28]. Consistent with this view, patients with higher miR-223 results in a worse survival, whereas lower level shows the opposite. However, in breast cancer, miR-223 has a negative correlation with the survival of patients [29]. Therefore, more studies are needed to elucidate the role of miR-223 in other cancers.

In conclusion, our results suggest selective expression of hnRNPK in a subset of PDAC cells may confer disease progression via enhancing transcription and activation of miR-223 in FBXW7-dependent manner. The hnRNPK/miR-223/FBXW7 feedback loop may represent a previously uncharacterized mechanism in PDAC cancer patients. Targeted disruption of the hnRNPK/miR-223/FBXW7 feedback loop may have therapeutic implications to improve clinical outcomes in patients with advanced PDAC.

## MATERIALS AND METHODS

### Cell culture and antibodies and reagents and transfection

The certified human PDAC cell lines Aspc-1, Panc-1, SW1990, Hs766T, BxPC-3, Capan-2 and Capan-1 were purchased from the American Type Culture Collection (ATCC; Rockville, MD). These cells were cultured in phenol red free Dulbecco's Modified Eagle Medium (DMEM; Lonza, USA) or phenol red free RPMI-1640 medium (Gibco) containing 10% heat inactivated FCS, 2mM L-glutamine, 1mM sodium pyruvate, and 4.5g/l glucose (Sigma). All cell culture experiments were carried out without antibiotics.

The following antibodies were used for Western blot analysis: FBXW7 (Bethyl), GSK3β (Cell Signaling), HA (6E2, Cell Signaling), ubiquitin (P4D1, Cell Signaling), Flag (M2, Sigma-Aldrich), and β-Actin (Cell Signaling), Tubulin (Sigma-Aldrich), HnRNPK ( Abcam). Mouse anti-GST antibody (SAB4200237) were purchased from Sigma. Mitomycin C, Z-Leu-Leu-Leu-al (MG132) was from Calbiochem. CHX (C4859), and puromycin (P8833) were from Sigma. The GSK3β inhibitor VIII was purchased from EMD Millipore. Transfections were performed in 35×10–mm tissue culture dishes by using 1.0 μg of DNA and 2.5 μl of Lipofectamine 2000 (Invitrogen). FBXW7 was obtained from Ambion, and GSK3β, which was obtained from Cell Signaling; were transfected at 25 nM using Lipofectamine RNAiMAX (Invitrogen). The siRNA target sequences used in this study are as follows: Control 5′-GGGTATCGACGATTACAAATT-3′; FBXW7-1 5′-GTGGAATGCAGAGACTGGAGA-3′; and FBXW7-2 5′-CGGGTGAATTTATTCGAAATT-3′. HnRNP K siRNA, 5′-GGAACAAGCATTTAAAAGA- 3′ and 5′-TCTTTTAAATGCTTGTTCC- 3′. Lentiviruses bearing hnRNPK shRNAs were purchased from GeneChem (China). miR-223 antagomir sequences: 5′-GGGGTATTTTAGAACTGACA- 3′.

### Quantitative real-time PCR and plasmids and chromatin immunoprecipitation

Total RNA was isolated using TRIzol reagent and miRNeasy Mini Kits (Qiagen) according to the manufacturer's instructions. RNA concentration and purity were measured using a Nanodrop spectrophotometer (Thermo Fisher Scientific Inc., Germany). The specific RT primers used were: miR-223:5′-GTCGTATCCAGTGCGTGTCGTGGAGTCGGCAATTGCACTGGATACGACAACTCA-3′ and U6:5′-CGCTTCACGAATTTGCGTGTCA-3′. RT was performed using PrimeScript™ RT reagent Kit (Takara, Japan) according to the manufacturer's instructions. PCR primers used were: miR-223 sense, 5′-CAGAAAGCCCAATTCCATCT-3′ and antisense, 5′-GGGCAAATGGATACCATACC-3′; U6 sense, 5′-CTCGCTTCGGCAGCACA-3′ and antisense, 5′-AACGCTTCACGAATTTGCGT-3′; FBXW7 Forward, 5′-GTCCCGAGAAGCGGTTTGATA-3′; Reverse, 5′-TGCTCAGGCACGTCAGAAAAG-3′; GAPDH sense, 5′-GCACCGTCAAGGCTGAGAAC-3′ and antisense, 5′-TGGTGAAGACGCCAGTGGA-3′. QRT-PCR was performed using SYBR Premix ExTaq (TaKaRa, China) according to the manufacturer's protocol. GSK3β expression vector plasmid HA-ubiquitin (HA-Ub) plasmid and FBXW7 plasmids were kindly provided by Dr. Zhou (Rutgers University). Chromatin immunoprecipitation was performed as described previously [10].

### Luciferase reporter assay

A FBXW7-3′UTR luciferase reporter was created. Briefly, the 3′UTR sequence of FBXW7 predicted to interact with miR-223 was amplified and cloned into the EcoRI and XhoI sites of pGL3-luc vector (Promega, USA). The site-directed mutagenesis of the miR-223 target-site was carried out using Invitrogen (Californlia, USA). The constructs were sequenced and named pGL3-luc-FBXW7/3′-UTR-wt or pGL3-luc-FBXW7/3′-UTR-mut. For reporter assays, PADC cells were cultured in 24-well plates and each transfected with 100 ng of pGL3-luc-FBXW7/3′UTR-wt or pGL3-luc-FBXW7/3′UTR-mut and miR-223 mimics or inhibitor using Lipofectamine 2000 (Invitrogen, USA). 48 hours after transfection, cells were harvested and assayed with Dual-Luciferase Reporter Assay kit (Promega, USA) according to the manufacturer's instructions.

### PDAC patients’ samples

All tissues enrolled in this study were collected from patients undergoing PDAC resection from January 2007 to May 2013 at the Baoan Hospital of Southern Medical University. A total of 60 PDAC specimens, along with 20 normal pancreatic tissue (NPT) specimens, were obtained for immunohistochemistry (IHC) and *in situ* hybridization (ISH) studies. Hematoxylin-and eosin-stained slides were obtained for each samples. All pathology findings of the specimens were scored by two pathologists who were blinded to the subjects. Ethical approval for the use of human subjects was obtained from the Research Ethics Committee of the Baoan Hospital of of Southern Medical University with the informed consent of patients.

### *In situ* hybridization

ISH was performed using antisense oligonucleotide probes for miR-223 (Exiqon, USA), with scrambled-miR (5′-GTGTAACACGTCTATACGCCCA-3′) serving as a negative control. After the sections were deparaffinized, hydrated and deproteinated, prehybridization was performed in hybridization buffer for 2 h in a humidified chamber at 55°C. Hybridization was then performed by applying 20 nm of probe in hybridization buffer to the slides covered with nescofilm overnight at 55°C in a humidified chamber. Hybridized probes were detected by incubation with anti-digoxigenin–alkaline phosphatase conjugate at 37°C for 30 min, followed by substrate 3,3′-diaminobenzidine to develop a brown color. Finally, the cells were counterstained with methyl green for 3–5 min and mounted on slides.

Wound healing and immunohistochemical staining and MTT and colony forming assays.

These assays were performed as described previously [11].

### Ubiquitination assay

Indicated cells were transfected with the indicated plasmids. The cells were treated with MG132 for 6 h, harvested, and lysed with RIPA buffer supplemented with 10 mM iodoacetamide (GE Healthcare) and protease inhibitors. Endogenous hnRNPK was immunoprecipitated with 1 μg of anti-hnRNPK polyclonal antibody and immunoblotted with anti-HA or indicated antibodies. Or a 100 μL sample of the *in vitro* translated FLAG-tagged FBXW7 was purified using Flag beads, and the reconstituted complex was used for *in vitro* ubiquitination without elution from the Flag beads. 2 μL *in vitro* translated hnRNPK was incubated in a 30 μL reaction mixture containing 5 mM MgCl2, 0.6 mM DTT, 2 mM ATP, 50 mM Tris·HCl (pH 7.5), 100ng of ubiquitin-activating enzyme E1, 200 ng of ubiquitin-conjugating enzyme UbcH5c, 10 μg of ubiquitin (Calbiochem), and ~2 μg of FBXW7. After incubation at 37°C for 2 h, the reactions were boiled and separated on SDS/PAGE gel.

### Flow cytometry

The assay was performed according to the manufacturer's instructions. Cells were treated as indicated, harvested, washed with phosphate-buffered saline (PBS; Sigma-Aldrich, P4417). Analysis of cell death was performed using the Annexin V.Briefly, cells were suspended in ANXA5 binding buffer (10 mM HEPES, pH 7.4, 2.5 mM CaCl_2_, 140 mM NaCl) and stained with ANXA5 (BD, 561209). Samples were analysed on the FACSCanto^TM^ II Flow Cytometer (BD Biosciences). FACS Diva Software (BD Biosciences) was used to calculate the percentage of cells positive for annexin V and propidium iodide (PI). Both annexin V and PI negative staining represents viable cells, early apoptotic cells were positive for annexin V staining, both annexin V and PI positive staining means late apoptosis, but necrotic cells were positive for PI staining.

### Immunofluorescence

Indicated HCC cells (5 × 104 cells) were fixed with 4% paraformaldehyde, permeabilized with 0.1% Triton X-100 in PBS, and blocked with 3% BSA in PBS. Expression of E-cadherin and vimentin was detected using each respective primary antibody and visualized with Alexa Fluor 488-conjugated secondary antibodies (Invitrogen, USA). Nuclei were counterstained with Hoechst 33258. All scale bars represent 200 μm. All images were observed by DeltaVision RT wide-field epifluorescence microscope imaging system and softWoRxs image analysis program (Applied Precision, USA).

### Western blotting and co-immunoprecipitation

Cells were lysed in ice-cold RIPA buffer (phosphate-buffered solution containing 1% Nonidet P-40, 0.1% SDS and 0.5% sodium deoxycholate) supplemented with 50 mM NaF, 1 mM Na_3_VO_4_, 10 mM Na_4_P_2_O_7_, 5 μg ml^−1^ aprotinin, 5 μg ml^−1^ leupeptin and 1 mM PMSF. After the insoluble fraction was removed by centrifugation at 4°C for 10 min at 12,000 r.p.m., the whole-cell lysates were pre-cleared using protein-A/G sepharose. An equal amount of normal IgG was used as negative control. By incubating the above lysates with protein-G sepharose that was pre-absorbed with 2 μg of the indicated primary antibodies at 4°C overnight, immunoprecipitation was performed. After extensive washing, the sepharose beads were boiled in 50 μl of 1 × SDS–polyacrylamide gel electrophoresis loading buffer for 5 min at 95°C. The eluted proteins were then subjected to western blotting.

### Cell migration and invasion assay

Cell migration and invasion were determined by wound healing assay and invasion chamber assay, respectively as described previously [11].

### Cell cycle analysis

Indicated PDAC cells were harvested 72 h after indicated treatment. Then the cells were fixed in 75% ethanol overnight at −20°C after washed with PBS. Then the cells were washed with PBS for twice and applied with DNA Prep Stain (Beckman Coulter, USA) and RNase for 30 min. Next, the cell cycle analysis was performed by Flow Cytometry System with FACSDiva software. The data were analyzed by ModFit LT 3.2 software (Verity Software House, USA) and the cell cycle distribution was described as the percentage of cells in G1, S, and G2 populations.

### Xenograft mice model

All experiments involving animals were undertaken in accordance with the National Institute of Health Guide for the Care and Use of Laboratory Animals and with the approval of the Scientific Investigation Board of the Hospital. Balb/c athymic nude mice were treated as described previously [12].

### Statistical analysis

All data were analyzed using the statistical software SPSS 18.0 for Windows (SPSS Inc., IL). The DFS and OS rates were calculated using the Kaplan-Meier method, with the log-rank test applied for comparison. Spearman's rank analysis was used to analyze the correlations between different protein expressions level. The Student's *t*-test, χ^2^ test or Fisher's exact test was used for comparisons between groups. Variables with a value of *p* < 0.05 in univariate analysis were used in subsequent multivariate analysis on the basis of Cox regression analyses. Two-sided *p*-values were calculated, and statistically significant data are indicated by asterisks *P* < 0.05 (*), *P* < 0.01(**), *P* < 0.01(***). The results are expressed as mean ± standard deviation (S.D.) from at least three independent experiments.

## SUPPLEMENTARY MATERIALS FIGURES


