# Supplementary material for: HnRNPK/miR-223/FBXW7 feedback cascade promotes pancreatic cancer cell growth and invasion
Source: Oncotarget. 2017 Feb 20;8(12):20165–78. doi: 10.18632/oncotarget.15529 (PMC5386752; doi:10.18632/oncotarget.15529)
Supplement: Supplementary file 1 [file oncotarget-08-20165-s001.pdf]

# HnRNPK/miR-223/FBXW7 feedback cascade promotes pancreatic cancer cell growth and invasion

## SUPPLEMENTARY FIGURE

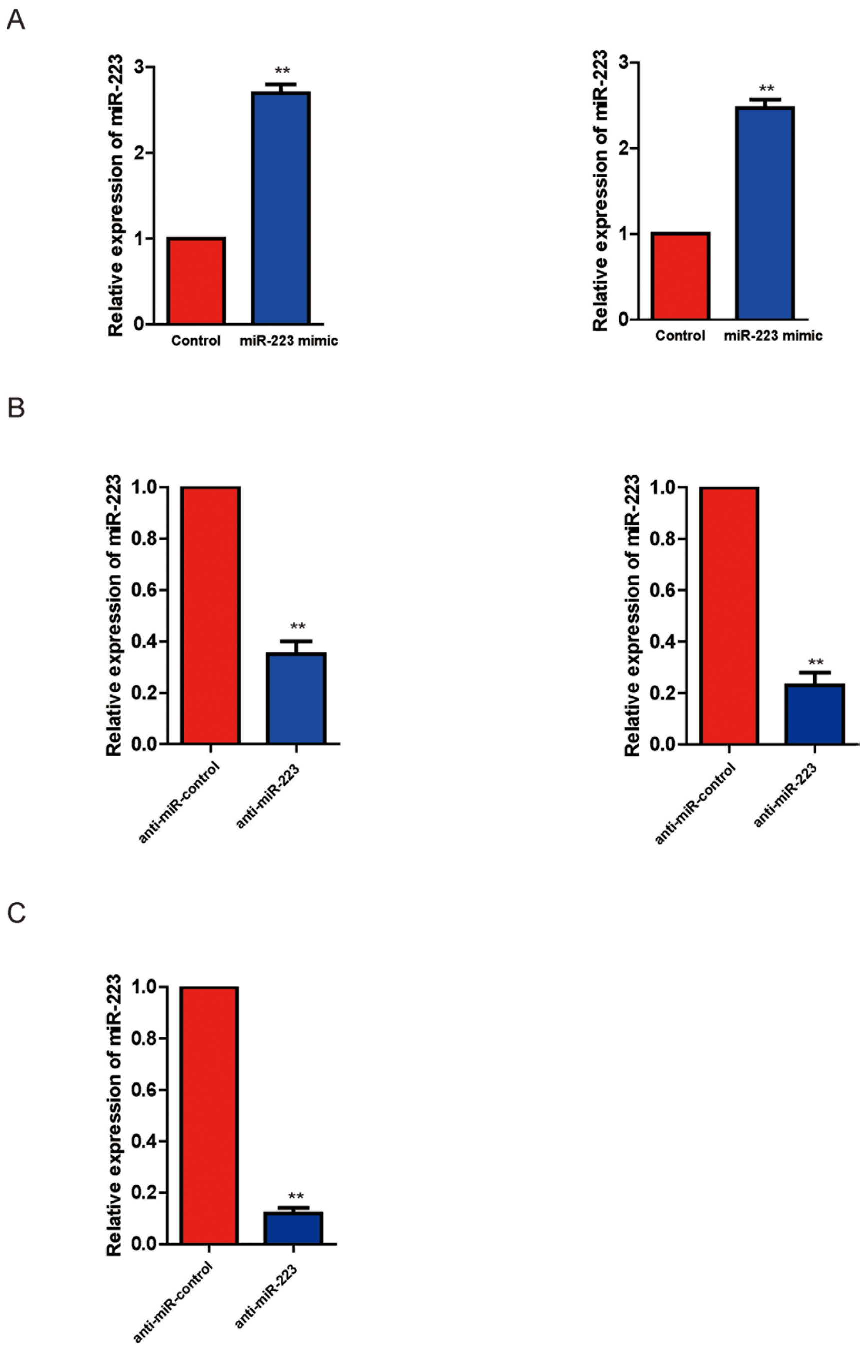


**Supplementary Figure 1: The change inrelative levels of miR-223 in PDAC cells A and B. or tissues C. treated as indicated.**
